# Association of maternal risk factors with fetal aneuploidy and the accuracy of prenatal aneuploidy screening: a correlation analysis based on 12,186 karyotype reports

**DOI:** 10.1186/s12884-023-05461-4

**Published:** 2023-03-02

**Authors:** Lun Wei, Jiakai Zhang, Ningxian Shi, Chao Luo, Le Bo, Xuanping Lu, Shasha Gao, Caiping Mao

**Affiliations:** 1 Reproductive Medicine Center, First Affiliated Hospital of Soochow University, No.899 Pinghai Road, Suzhou, 215006 Jiangsu China; 2 Marxism Research Institute, Soochow University, Suzhou, 215123 Jiangsu China; 3 Suzhou High School Affiliated to Xi’an Jiaotong University, Suzhou, Jiangsu China; 4 Department of Gynecology and Obstetrics, Sihong County People’s Hospital, Suqian, Jiangsu China

**Keywords:** Adolescent pregnancy, Fetal aneuploidy, IVF-ET, Maternal factors, NIPT, Prenatal aneuploidy screening

## Abstract

**Background:**

NIPT is becoming increasingly important as its use becomes more widespread in China. More details are urgently needed on the correlation between maternal risk factors and fetal aneuploidy, and how these factors affect the accuracy of prenatal aneuploidy screening.

**Methods:**

Information on the pregnant women was collected, including maternal age, gestational age, specific medical history and results of prenatal aneuploidy screening. Additionally, the OR, validity and predictive value were also calculated.

**Results:**

A total of 12,186 analysable karyotype reports were collected with 372 (3.05%) fetal aneuploidies, including 161 (1.32%) T21, 81 (0.66%) T18, 41 (0.34%) T13 and 89 (0.73%) SCAs. The OR was highest for maternal age less than 20 years (6.65), followed by over 40 years (3.59) and 35–39 years (2.48). T13 (16.95) and T18 (9.40) were more frequent in the over-40 group (*P < 0.01*); T13 (3.62/5.76) and SCAs (2.49/3.95) in the 35–39 group (*P < 0.01*). Cases with a history of fetal malformation had the highest OR (35.94), followed by RSA (13.08): the former was more likely to have T13 (50.65) (*P < 0.01*) and the latter more likely to have T18 (20.50) (*P < 0.01*). The sensitivity of primary screening was 73.24% and the NPV was 98.23%. The TPR for NIPT was 100.00% and the respective PPVs for T21, T18, T13 and SCAs were 89.92, 69.77, 53.49 and 43.24%, respectively. The accuracy of NIPT increased with increasing gestational age (0.81). In contrast, the accuracy of NIPT decreased with maternal age (1.12) and IVF-ET history (4.15).

**Conclusions:**

①Pregnant patients with maternal age below 20 years had higher risk of aneuploidy, especially in T13; ②A history of fetal malformations is more risky than RSA, with the former more likely to have T13 and the latter more likely to have T18; ③Primary screening essentially achieves the goal of identifying a normal karyotype, and NIPT can accurately screen for fetal aneuploidy; ④A number of maternal risk factors may influence the accuracy of NIPT diagnosis, including older age, premature testing, or a history of IVF-ET. In conclusion, this study provides a reliable theoretical basis for optimizing prenatal aneuploidy screening strategies and improving population quality.

## Background

Fetal aneuploidy is often associated with disease and developmental abnormalities that severely affect the health and quality of life of patients and place a heavy burden on families and society, most notably T21, T18, T13 and SCAs. Current prenatal primary screening follows the sequential screening risk assessment method proposed in 1999 [1], which combines NT scan, ultrasound and serological screening in early/mid pregnancy to provide clinicians with as much information as possible. NIPT is a method of sequencing free fetal DNA fragments in maternal plasma using massively parallel sequencing technology, which provides excellent detection of common fetal aneuploidies [2]. Clear risk factors and effective screening strategies can effectively prevent fetal aneuploidy, reduce the incidence of birth defects and improve the quality of the population. This study was a retrospective analysis of 12,186 karyotype reports. Maternal risk factors for fetal aneuploidy were investigated and the clinical value of prenatal aneuploidy screening was assessed.

## Methods

### Research objects

This study collected information on pregnant women with singleton pregnancies who underwent amniocentesis, cordocentesis (amniocentesis could not be performed due to high gestational age), or chromosomal testing of aborted tissue at the First Affiliated Hospital of Soochow University, Jiangsu Province, China and Sihong County People’s Hospital, Jiangsu Province, China from February 2018 to January 2022. Exclusion criteria included: pregnant women with body mass index less than 18.5 kg/m^2^ or greater than 23.9 kg/m^2^, use of prohibited or cautionary medications before and during pregnancy, a spouse with a clear abnormality in chromosome number or structure, incomplete information, malignancy, and a history of allogeneic blood transfusion, transplantation, stem cell therapy, immunotherapy, or radiation exposure within the 3 months before pregnancy. All samples were anonymized and did not influence or adversely affect the final pregnancy outcome. The study was approved by the Ethics Committee of the First Affiliated Hospital of Soochow University, and a waiver of informed consent was granted for this study with approval number 2021(325). We confirm that all methods were performed in accordance with relevant guidelines and regulations.

### Data processing

Information was collected on maternal age, gestational age, medical history (including IVF-ET, RSA and fetal malformations) and prenatal aneuploidy screening results (including primary screening and NIPT). Prior to the logistic regression analysis, four groups were created: under 20 years, 20–34 years, 35–39 years and 40 years and older, with 20–34 years being the unexposed group and the remaining groups being the exposed group. Similarly, cases with unremarkable medical history were included in the unexposed group and cases with a history of IVF-ET, RSA and fetal malformations were included in the exposed group. The incidence of each type of fetal aneuploidy under different maternal risk factors was counted based on karyotype reports of fetal or aborted tissue.

In this study, the risk levels for prenatal aneuploidy screening and NIPT were determined according to relevant guidelines and expert consensus in mainland China. In short, all pregnant women should undergo primary screening and NIPT is not necessary for all. The decision on the need for prenatal diagnosis is also based on these results. It is important to emphasize that the doctor only provides guidance and advice throughout the pregnancy and that the pregnant woman has complete independence of choice. For primary screening, any of the following is considered high risk: NT < 2.5–3.0 mm in early pregnancy, abnormal fetal growth parameters throughout pregnancy and a Down’s screening result above 1/270 of the cut-off (biochemical markers and algorithms are being introduced to estimate risk, including AFP, total hCG, unconjugated estriol and free beta-hCG). For NIPT, free fetal DNA fragments purified from maternal peripheral plasma are sequenced using DNA sequencing technology, and the results are subjected to data processing and bioinformatic analysis. The NIPT is considered high risk if the detection risk index exceeds a threshold of 3, otherwise it is considered low risk. Based on the results of primary screening and NIPT, karyotypes were counted under different screening results.

### Statistical analysis

In the study of the correlation between maternal age and fetal aneuploidy, 20–34 years was defined as the unexposed group. Binary logistic regression analysis was first used to analyze the association between aneuploidy and each exposed group and to calculate the correlation strength index OR with the relevant statistical parameters. Multiple logistic regression analysis was then used to further analyze the association between different aneuploidy types (including T21, T18, T13 and SCAs) and each exposure group. In the correlation study between medical history and fetal aneuploidy, the unremarkable medical history was defined as the unexposed group and the analysis procedure was as above.

For the clinical evaluation of prenatal aneuploidy screening, we assessed indicators of validity (including TPR, TNR, FPR, FNR, Jordan index) and indicators of predictive value (including PPV and NPV). Further statistics on the accuracy of primary screening and NIPT were based on karyotype reports. Binary logistic regression was then used to analyze the correlation between maternal factors, gestational week of testing and screening accuracy. Maternal age (in years) and gestational age (in weeks) were used as measures in the statistical analysis. In the analysis of medical history, the unremarkable medical history was defined as the unexposed group.

Excel 16.6 (Microsoft Corporation, released 2016) was used for data collection and enumeration data were expressed as frequencies, proportions or constituent ratios. SPSS 22.0 (IBM, released 2019) was used for statistical analysis. Prism 9.0 (Graphpad, Inc., released in 2020) was used to visualize the statistical results.

## Results

### General statistical description

**Fig. 1:**
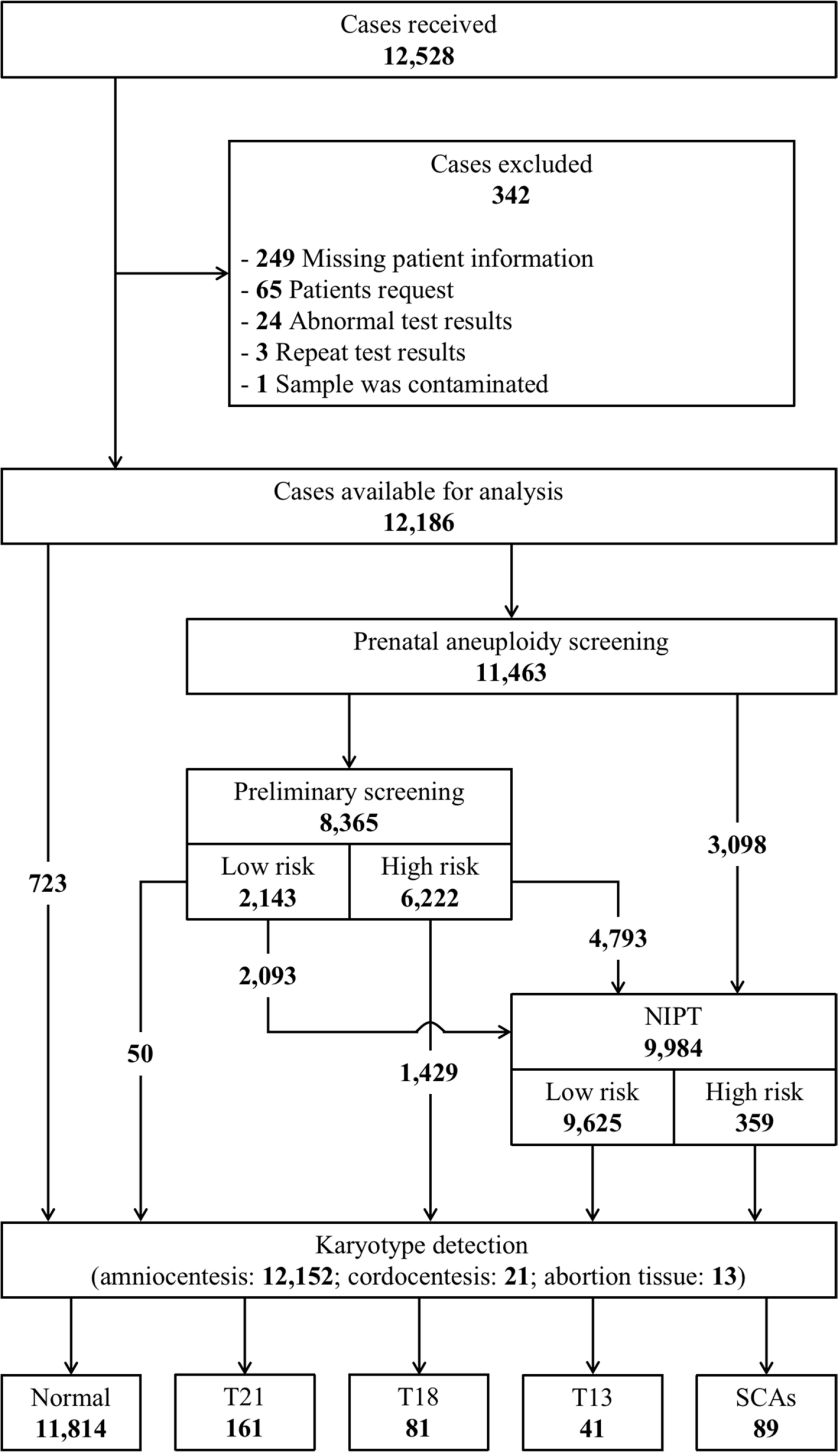
The flow chart showing the study identification and selection process

A total of 12,528 karyotype reports were collected for this study and 342 were excluded for a variety of reasons including missing patient information, patient request, abnormal test results, duplicate test results and sample contamination. A total of 12,186 analyzable reports were obtained. Of these, 11,463 cases underwent prenatal aneuploidy screening (primary screening and/or NIPT) and 723 patients did not undergo prenatal aneuploidy screening. A total of 8365 cases underwent primary screening and 9984 cases underwent NIPT: 71 cases (0.58%) with maternal age under 20 years; 9128 cases (74.91%), 20–34 years; 2298 cases (18.86%), 35–39 years; 689 cases (5.65%), over 40 years (Fig. 1).

Two thousand three hundred sixty-five cases (19.41%) underwent NIPT before 16^+ 6^ gestation week; 3633 cases (29.81%) underwent NIPT in 17^+ 0^–19^+ 6^ gestation week; 3697 cases (30.34%) underwent NIPT in 20^+ 0^–22^+ 6^ gestation week; 2491 cases (20.44%) underwent NIPT after 23^+ 0^ gestation week; 11,804 cases (96.87%) were spontaneous pregnancies and 382 cases (3.13%) were IVF-ET cases. There was a history of RSA in 362 cases (2.97%) and fetal malformations in 92 cases (0.75%). The final karyotype was normal in 11,814 cases (96.95%), T21 in 161 cases (1.32%), T18 in 81 cases (0.66%), T13 in 41 cases (0.34%) and SCAs in 89 cases (0.73%) (Table 1).

**Table 1:** Statistics of general clinical characteristics of 12,186 karyotype reports

Charactristics	Cases (n)	Percentage (%)
**Maternal age (years old)**		
< 20	71	0.58
20–34	9128	74.91
35–39	2298	18.86
≥40	689	5.65
**Gestational age (weeks)**		
≤16^+ 6^	2365	19.41
17^+ 0^–19^+ 6^	3633	29.81
20^+ 0^–22^+ 6^	3697	30.34
≥23^+ 0^	2491	20.44
**Method of conception**		
Natural conception	11,804	96.87
IVF-ET	382	3.13
**History of RSA**		
Yes	362	2.97
No	11,824	97.03
**History of fetal malformation**		
Yes	92	0.75
No	12,094	99.25
**Prenatal screening situation**		
Preliminary screening alone	1479	12.14
NIPT alone	3098	25.42
Preliminary screening and NIPT	6886	56.51
No screening	723	5.93
**Preliminary screening**		
High-risk	6222	51.06
Low-risk	2143	17.59
Not done	3821	31.35
**NIPT**		
High-risk	359	2.95
Low-risk	9625	78.98
Not done	2202	18.07
**Method of karyotype detection**		
Amniocentesis	12,152	99.72
Cordocentesis	21	0.17
Abortion tissue	13	0.11
**Karyotype results**		
Normal	11,814	96.95
T21	161	1.32
T18	81	0.66
T13	41	0.34
SCAs	89	0.73

Preliminary screening combines the NT scanning, ultrasound and serological screening; NIPT: noninvasive prenatal testing; T21: trisomy 21/Down’s syndrome; T18: trisomy 18/Edwards’ syndrome; T13: trisomy 13/Patau’s Syndrome; SCAs: sex chromosome aneuploidies

### Effects of maternal risk factors on fetal aneuploidy

**Fig. 2:**
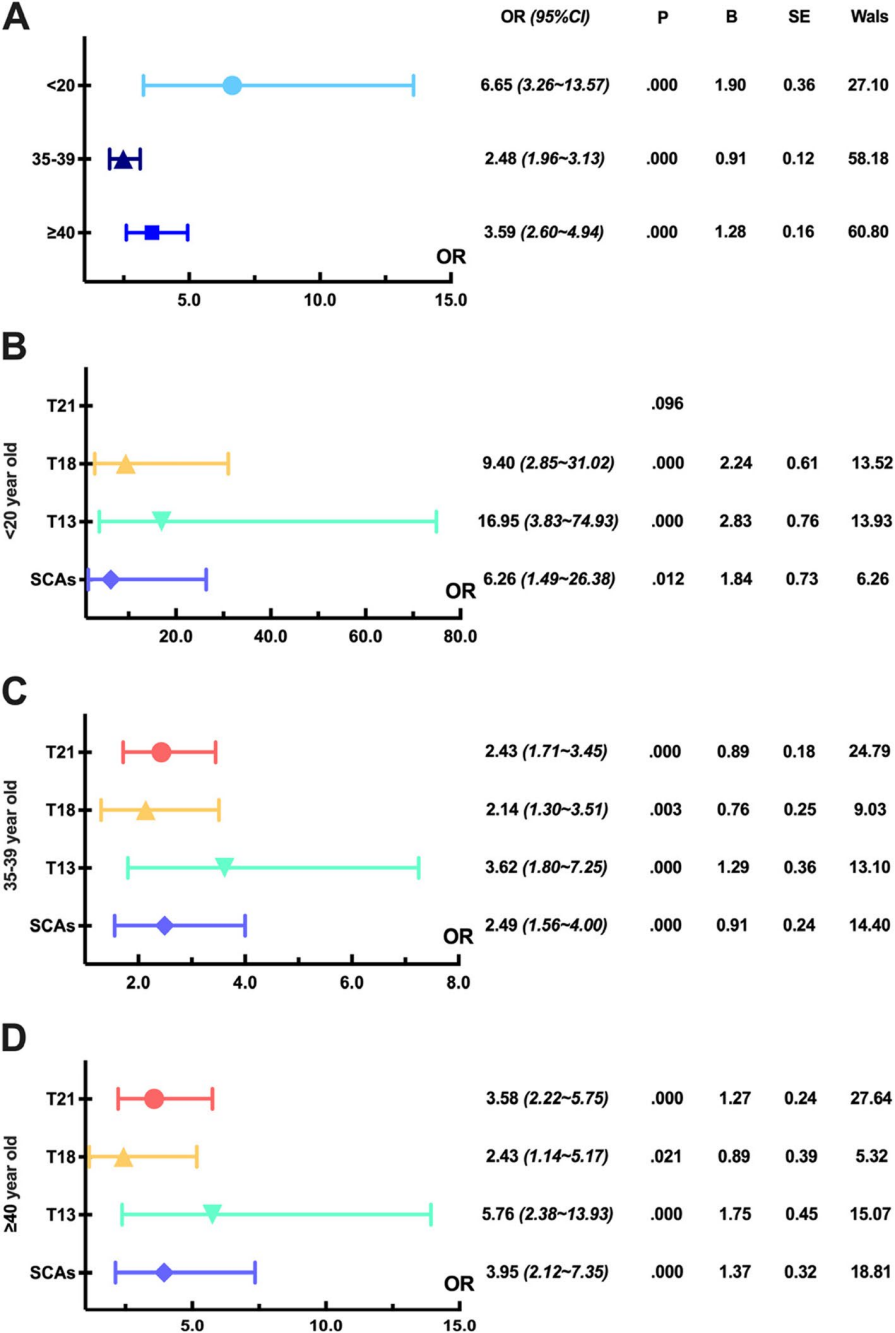
Analysis of correlation strength between fetal aneuploidy and maternal age factors

Analyzing the effect of maternal age on fetal aneuploidies, the highest OR was found with maternal age under 20 years (6.65), followed by over 40 years (3.59) and 35–39 years (2.48) (Fig. 2A). In addition, cases with mothers younger than 20 years had a higher risk of T13 (16.95) and T18 (9.40) (*P < 0.01*), whereas there was no statistical difference in T21 compared to 20–34 years (*P > 0.05*) (Fig. 2B). Those aged 35–39 years had a higher risk of T13 (3.62) and SCAs (2.49) (*P < 0.01*) (Fig. 2C). Those aged over 40 years had a higher risk of T13 (5.76) and SCAS (3.95) (*P < 0.01*) (Fig. 2D).

**Fig. 3:**
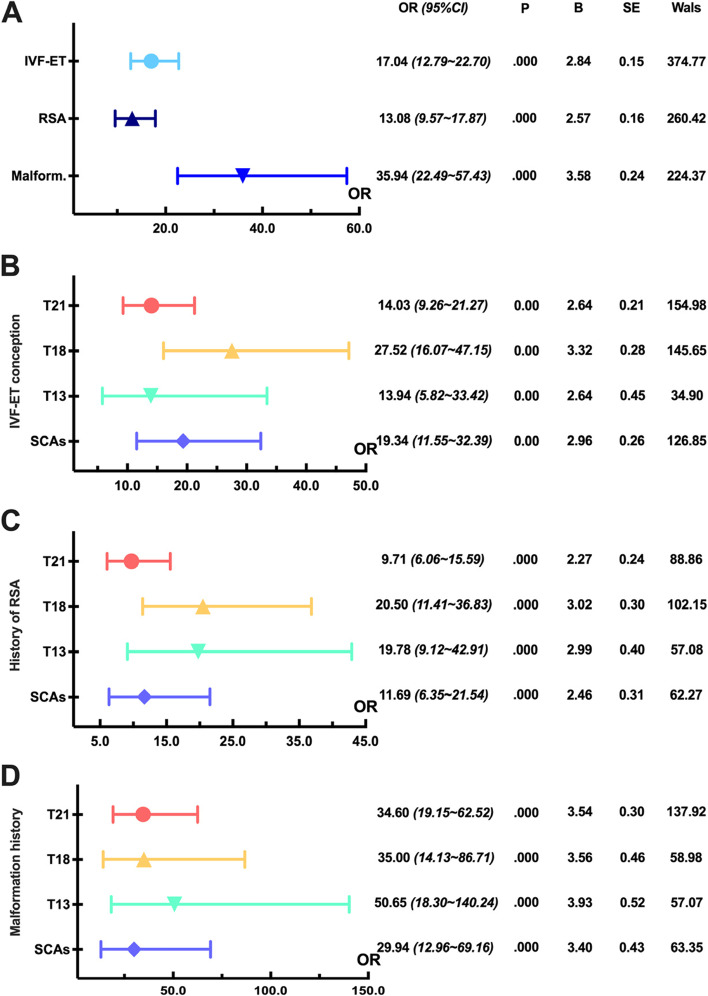
Analysis of correlation strength between fetal aneuploidy and maternal special medical history

Analyzing the effect of history on fetal aneuploidy, we found the highest OR for fetal malformation history (35.94), followed by IVF-ET history (17.04) and RSA (13.08) (*P < 0.01*) (Fig. 3A). In addition, the risk of T18 (27.52) and SCAs (19.34) was higher for IVF-ET history (*P < 0.01*) (Fig. 3B), while the risk of T18 (20.50) and T13 (19.78) was higher for RSA history (*P < 0.01*) (Fig. 3C), and the risk of T13 (50.65) and T18 (35.00) was higher for fetal malformation history (*P < 0.01*) (Fig. 3D). Table 2 shows the maternal risk factor statistics for 12,186 karyotype reports.

**Table 2:** Statistics of maternal risk factors for 12,186 karyotype reports

Karyotype	Maternal age (years old) [n] (%)				Medical history [n] (%)				Total [n] (%)
	< 20	20–34	35–39	≥40	Unremarkable	IVF-ET	RSA	Malformations	
**Normal**	62 (87.3)	8933 (97.9)	2180 (94.9)	639 (92.7)	11,164 (98.4)	295 (77.2)	297 (82.0)	58 (63.0)	11,814 (96.9)
**Abnormal**	9 (12.7)	195 (2.1)	118 (5.1)	50 (7.3)	186 (1.6)	87 (22.8)	65 (18.0)	34 (37.0)	372 (3.1)
**T21**	2 (22.2)	86 (44.1)	51 (43.2)	22 (44.0)	89 (47.8)	33 (37.9)	23 (35.4)	16 (47.1)	161 (43.3)
**T18**	3 (33.3)	46 (23.6)	24 (20.3)	8 (16.0)	33 (17.7)	24 (27.6)	18 (27.7)	6 (17.6)	81 (21.8)
**T13**	2 (22.2)	17 (8.7)	15 (12.7)	7 (14.0)	19 (10.2)	7 (8.0)	10 (15.4)	5 (14.7)	41 (11.0)
**SCAs**	2 (22.2)	46 (23.6)	28 (23.7)	13 (26.0)	45 (24.2)	23 (26.4)	14 (21.5)	7 (20.6)	89 (23.9)
**Total**	71	9128	2298	689	11,350	382	362	92	12,186

*T21* trisomy 21/Down’s syndrome, *T18* trisomy 18/Edwards’ syndrome, *T13* trisomy 13/Patau’s Syndrome, *SCAs* sex chromosome aneuploidies, IVF-ET in vitro fertilization and embryo transfer, *RSA* recurrent spontaneous abortion

### Clinical evaluation of prenatal aneuploidy screening

Of the 11,463 cases that underwent prenatal aneuploidy screening (primary screening and/or NIPT), a total of 8365 cases underwent primary screening and 9984 cases underwent NIPT. After primary screening, 6222 high-risk cases and 2143 low-risk cases were reported. After NIPT, 359 high-risk and 9625 low-risk cases were reported. Of these, 238 karyotype abnormalities (66.30%) were reported in the high-risk group and 9625 karyotypes (100.00%) were reported in the low-risk group (Table 3).

**Table 3:** Statistics of the information and reports for prenatal aneuploidy screening

Karyotype	Primary screening [n] (%)		NIPT [n] (%)		Nothing [n] (%)
	High-risk	Low-risk	High-risk	Low-risk	
**Normal**	6118 (98.33)	2105 (98.23)	121 (33.70)	9625 (100.00)	642 (88.80)
**Abnormal**	104 (1.67)	38 (1.77)	238 (66.30)	0 (0.00)	81 (11.2)
**T21**	46 (44.23)	21 (55.26)	107 (44.96)	0 (0.00)	37 (45.68)
**T18**	23 (22.12)	10 (26.32)	60 (25.21)	0 (0.00)	9 (11.11)
**T13**	8 (7.69)	2 (5.26)	23 (9.66)	0 (0.00)	11 (13.58)
**SCAs**	27 (25.96)	5 (13.16)	48 (20.17)	0 (0.00)	24 (29.63)
**Total**	6222	2143	359	9625	723

*T21* trisomy 21/Down’s syndrome, *T18* trisomy 18/Edwards’ syndrome, *T13* trisomy 13/Patau’s Syndrome, *SCAs* sex chromosome aneuploidies

We then calculated the validity and predictive values to assess primary screening and NIPT. The sensitivity of primary screening was 73.24%, the specificity was 25.60%, and the PPV and NPV were 1.67 and 98.23%, respectively. The total TPR, TNR, PPV and NPV of NIPT were 100.00, 98.76, 66.30 and 100.00%, respectively. In addition, NIPT achieved 100.00% TPR and NPV, 99.88, 99.73, 99.79 and 99.35% TNR and 89.92, 69.77, 53.49 and 43.24% PPV for T21, T18, T13 and SCAs, respectively (Table 4).

**Table 4:** Statistics of the authenticity indicator and predictive value for primary aneuploidy screening and NIPT

Prenatal screening	TP [n]	FP [n]	FN [n]	TN [n]	TPR (%)	TNR (%)	Accuracy (%)	Youden’s index	FPR (%)	FNP (%)	PPV (%)	NPV (%)
**Preliminary**	104	6118	38	2105	73.24	25.60	49.42	−0.0116	74.40	26.76	1.67	98.23
**NIPT**	238	121	0	9625	100.00	98.76	99.38	0.9876	1.24	0.00	66.30	100.00
**T21**	107	12	0	9734	100.00	99.88	99.94	0.9988	0.12	0.00	89.92	100.00
**T18**	60	26	0	9720	100.00	99.73	99.87	0.9973	0.27	0.00	69.77	100.00
**T13**	23	20	0	9726	100.00	99.79	99.90	0.9979	0.21	0.00	53.49	100.00
**SCAs**	48	63	0	9683	100.00	99.35	99.68	0.9935	0.65	0.00	43.24	100.00

*TPR* sensitivity/true positive rate, *TNR* specificity/true negative rate, *FPR* false positive rate, *FNR* false negative rate, *PPV* positive predictive value, *NPV* negative predictive value, *T21* trisomy 21/Down’s syndrome, *T18* trisomy 18/Edwards’ syndrome, *T13* trisomy 13/Patau’s Syndrome, *SCAs* sex chromosome aneuploidies

### Effects of maternal risk factors on screening accuracy

In 2209 cases (26.4%) the primary screening result was consistent with the karyotype report and in 6156 cases (73.5%) the primary screening result was inconsistent with the karyotype report. In 9863 cases (98.7%) the NIPT result was consistent with the karyotype report and in 121 cases (1.2%) the NIPT result was inconsistent with the karyotype report (Table 5).

**Table 5:** Statistics of maternal factors influencing the accuracy of prenatal aneuploidy screening

Impact factor	Consistency [n] (%)		Total [n]
	Yes	No	
**Primary screening**	2209 (26.4)	6156 (73.5)	8365
**Maternal age (years)**	Regarded as measurement data		
**Unremarkable**	2022 (25.7)	5826 (74.2)	7848
**IVF-ET**	84 (36.0)	149 (63.9)	233
**RSA**	79 (34.4)	150 (65.5)	229
**Malformations**	24 (43.6)	31 (56.3)	55
**NIPT**	9863 (98.7)	121 (1.2)	9984
**Maternal age (years)**	Regarded as measurement data		
**Gestational week (weeks)**	Regarded as measurement data		
**Unremarkable**	9111 (98.9)	100 (1.0)	9211
**IVF-ET**	345 (95.3)	17 (4.6)	362
**RSA**	335 (99.1)	3 (0.8)	338
**Malformations**	72 (98.6)	1 (1.3)	73

**Fig. 4:**
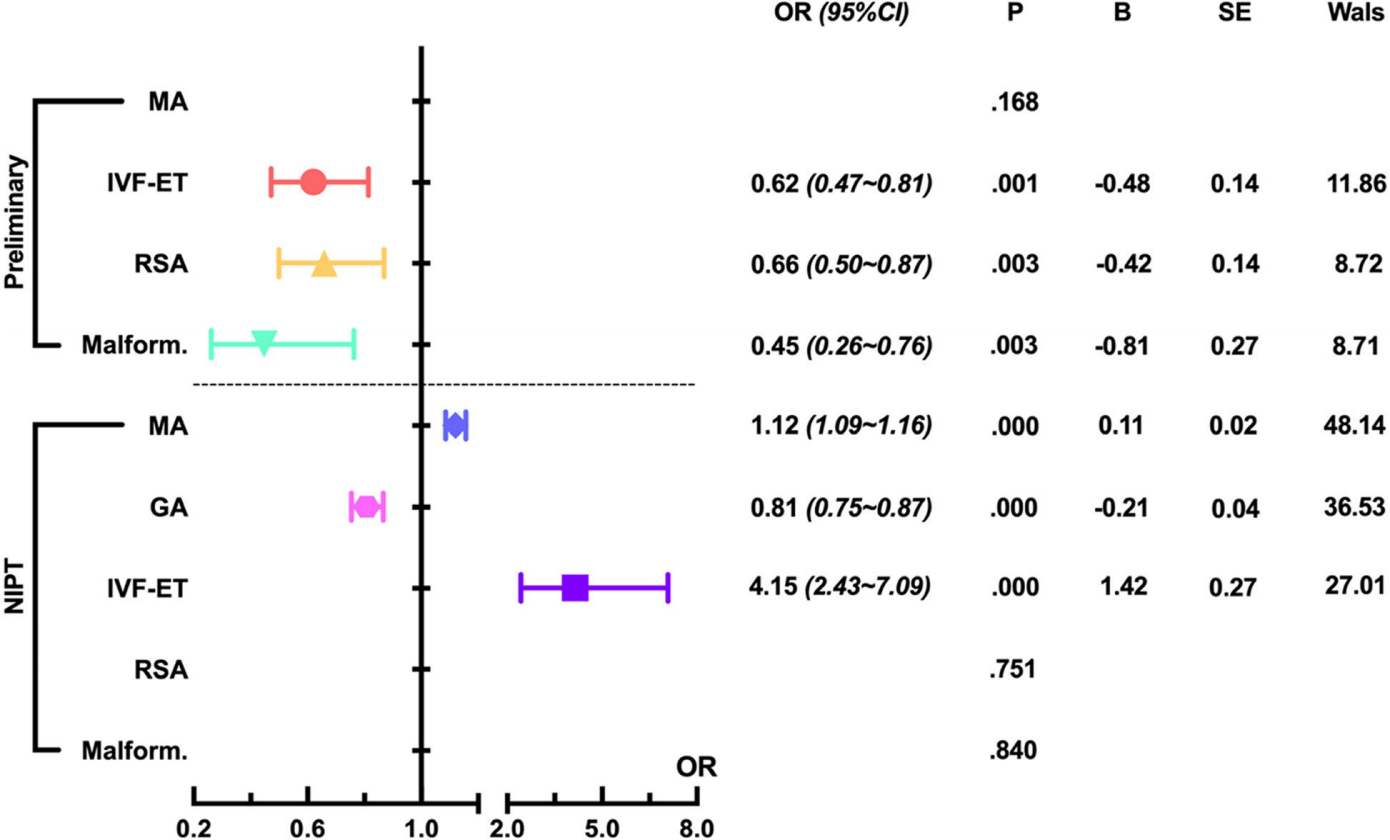
Analysis of correlation strength between the consistency of prenatal aneuploidy screening and maternal factors

To analyze the effect of potential maternal factors on the accuracy of primary screening, we found that a history of certain medical conditions improved the accuracy of primary screening (OR < 1). Further analysis showed that a history of fetal malformations had the lowest OR (0.45), followed by a history of IVF-ET (0.62) and RSA (0.66) (*P < 0.01*), while maternal age had no significant effect on primary screening results (*P > 0.05*). When analyzing the effect of potential maternal factors on NIPT accuracy, we found that NIPT accuracy decreased with increasing maternal age (1.12), whereas accuracy increased with increasing gestational age (0.81) (*P < 0.01*). History of IVF-ET (4.15) also decreased the accuracy of NIPT diagnosis, whereas RSA and fetal malformations had no significant effect on NIPT accuracy (*P > 0.05*) (Fig. 4).

## Discussion

Our results support an association between maternal age and fetal aneuploidy. It is well known that AMA (maternal age over 35 years) increases the risk of fetal aneuploidy [3, 4] and we also found that AMA is more likely to be associated with T13 and SCAs. It is important to note that T21 is the earliest identified and most easily understood human autosomal aberration and is the most easily identified in prenatal aneuploidy screening. Therefore, in actual clinical practice, many embryos or fetuses with T21 do not undergo chromosomal diagnosis. Therefore, our results do not prove that T13 and SCAs are more common in AMA than T21. Furthermore, the present study shows that adolescent pregnancies (maternal age < 20 years) seem to be riskier than AMA, especially in T13. Some studies have reported an association between adolescent pregnancies and adverse pregnancy outcomes, including preterm birth, miscarriage, high neonatal mortality, high infant mortality, high under-five mortality and fetal growth restriction [5–7], and other studies have reported that births to young mothers may be a marker of low fertility [8].

It was very difficult to collect samples under the age of 20 in this study as the legal age of marriage for women in mainland China is 20 and the principle of patient autonomy is respected in the consultation process. Therefore, we could not collect a larger amount of samples of teenage pregnancies. Also, we cannot force all pregnant women to undergo karyotyping of fetus or aborted tissue. This is a retrospective cohort study and we cannot intervene or give guidance beyond the principles of treatment. However, at least we observed some positive results in all samples that reported karyotyping. WHO estimates that adolescent pregnancies account for 11% of all births worldwide, with more than 95% of these occurring in developing countries [9]. This study provides a sound theoretical basis for reducing adolescent pregnancy, improving reproductive health and implementing public health interventions in low- and middle-income countries.

There is increasing evidence of an association between maternal risk factors and aneuploidy pregnancy loss [10, 11] and this study also supports an association between a history of specific medical conditions and fetal aneuploidy. Of the special medical histories collected, a history of fetal malformations was associated with a higher risk of aneuploidy and was more likely to have T13 and T18. A history of RSA was associated with a slightly higher risk of T18 than T13, whereas a history of IVF-ET was more likely to have T18 and SCAs. This finding is more easily explained by the fact that embryos with severe chromosomal abnormalities tend to stop growing early in pregnancy, whereas fetuses with chromosomal abnormalities but who are barely viable often have a variety of malformations or disorders [12, 13].

Notably, the findings of increased risk of fetal aneuploidy with IVF appear to be inconsistent with previous reports [14–16]. We conducted further follow-up and found that although fetal malformations or RSA could not be diagnosed prior to IVF, there must have been some specific reasons for choosing IVF, such as older maternal age, biochemical pregnancy or only one case of embryonic arrest, and untested aborted tissue. In addition to the IVF process itself, ovarian stimulation and cryopreservation are potential factors contributing to fetal aneuploidy [17, 18] and we must acknowledge that the aforementioned confounding factors were not controlled for in this study and it was difficult for us to do so. There is no doubt that the rapid development of ART has solved many fertility problems. However, as the technology has become more widespread, it has been found that most couples are more likely to achieve a normal chromosomal pregnancy without ART [19]. Although PGT-A can accurately detect most chromosomal abnormalities in embryos, we cannot force all couples undergoing IVF-ET to undergo PGT, which would have socio-economic and reproductive ethical implications. At the same time, there is still an urgent need for PGT to overcome some technical problems, such as the low efficiency of chimerism detection [20].

Prior to the discovery and popularization of NIPT, sequential screening by ultrasound combined with serology was the most effective screening method for prenatal chromosomal abnormalities. In this study, the TPR and NPV of primary screening were 73.24 and 98.23%, respectively, essentially achieving the goal of identifying normal karyotypes. For experimental results where specific medical history improved the accuracy of primary screening, we analyzed them in the context of practical clinical work and found that there was a greater degree of diagnostic suspicion bias. For example, sonographers will perform more detailed examinations on pregnant women with a specific medical history, leading to more reliable results.

Further combination of primary screening and NIPT methods has yielded greater health economic benefits [21, 22]. NIPT had high sensitivity and specificity in this study, with high PPV in T21 (89.92%) and T18 (69.77%) and moderate PPV in T13 (53.49%) and SCAs (43.24%), which is generally consistent with previous reports [23–26]. Apart from aneuploidy, NIPT has also made some breakthroughs in the diagnosis of chromosomal abnormalities such as monogenic diseases and copy number variants [27–30]. In conclusion, NIPT is of great social and economic value in accurately screening fetuses with trisomy and SCAs, thereby improving reproductive health [21, 31].

Due to the extremely low levels of cffDNA, the accuracy of NIPT analysis is highly dependent on the presence of sufficient cffDNA in the sample [32]. In this study, maternal age, early gestational age at NIPT testing or a history of IVF-ET were found to reduce the accuracy of the test, all of which were directly related to the cffDNA ratio in the peripheral blood. The cffDNA ratio has been reported to be negatively correlated with maternal age and positively correlated with gestational age [33, 34]. In addition, one study showed that cffDNA was reduced in ART patients compared to natural pregnancies and that cf. fdna was more significantly reduced in ART pregnancies after fresh ET than after frozen ET [35]. Recent studies have also found a significantly higher false positive rate in the ART population, especially for T13 and SCAs [36]. In addition to prenatal aneuploidy screening, the NIPT technique, based on plasma cell-free DNA testing, has been applied to cancer diagnosis and the assessment of immune rejection after transplantation [37].

Maternal occult tumors have been shown to cause NIPT results to be inconsistent with karyotype reports [38]. This raises concerns about the 121 NIPT results in our sample that were inconsistent with the karyotype report, although we did not follow up pregnant women with cancer. Primary care physicians should emphasize the significance and importance of cancer screening to pregnant women. Furthermore, NIPT has limited ability to identify sex and screen for chromosomal abnormalities in multiple pregnancies, and the efficiency of the test is inconsistent. Therefore, greater caution should be exercised in interpreting NIPT results in women with multiple pregnancies [39, 40].

To further improve the accuracy of NIPT in prenatal aneuploidy screening, many scholars have explored various aspects of bioinformatics technology and software engineering to improve the technology [41, 42]. Domestic scholars have found that cffRNA is more stable in maternal circulation [43]. Although NIPT has many clear advantages, the introduction of NIPT into routine antenatal care in many countries has also raised a number of ethical issues [31]. The debate has centered on the impact of prenatal aneuploidy screening on pregnancy outcomes and the fact that equal access to healthcare for every pregnant woman is a core principle in areas of self-paying NIPT [44, 45]. Therefore, the need for high-quality pregnancy counselling and a well-developed process for prenatal aneuploidy screening challenges the quality of maternal health services in each country [46, 47].

Finally, some limitations of this study need to be explained. Studies have shown an association between paternally inherited risk factors and adverse pregnancy outcomes [48]. Paternal information was not collected in this study, which may have confounded the results to some extent. The results of this study are not representative of the overall level of screening in mainland China. The service area mainly covers the whole of southern and northern Jiangsu province and parts of Anhui province. This is one of the most developed and modernized regions in mainland China. Again, the results of this study are not representative of the incidence of fetal aneuploidy in the region. The sample is influenced by the actual clinical practice and guidelines for prenatal aneuploidy screening and does not represent the findings of the whole or randomly selected population in the region.

## Conclusions

Our results reveal more details about maternal risk factors that influence fetal aneuploidy: ①Pregnant patients with maternal age below 20 years had higher risk of aneuploidy, especially in T13; ②A history of fetal malformations is more risky than RSA, with the former more likely to have T13 and the latter more likely to have T18. In addition, we conducted a clinical evaluation of prenatal aneuploidy screening: ③Primary screening essentially achieves the goal of identifying a normal karyotype, and NIPT can accurately screen for fetal aneuploidy; ④A number of maternal risk factors may influence the accuracy of NIPT diagnosis, including older age, premature testing, or a history of IVF-ET.

## Data Availability

The datasets used or analysed during the current study are available from the corresponding author on reasonable request.

## Abbreviations

AMA: Advanced Maternal Age
ART: Assisted Reproductive Technology
cffDNA: Cell-Free Fetal DNA
FN: False Negatives
FNR: False Negative Rate
FP: False Positives
FPR: False Positive Rate
IVF-ET: In Vitro Fertilization and Embryo Transfer
NIPT: Non-Invasive Prenatal Testing
NPV: Negative Predictive Value
NT: Nuchal Translucency
OR: Odds Ratio
PGT(−A): Preimplantation Genetic Testing (for Aneuploidy)
PPV: Positive Predictive Value
RSA: Recurrent Spontaneous Abortion
SCAs: Sex Chromosome Aneuploidies
T13: Trisomy 13 / Patau’s Syndrome
T18: Trisomy 18 / Edwards’ syndrome
T21: Trisomy 21 / Down’s syndrome
TN: True Negatives
TNR: True/specificity Negative Rate
TP: True Positives
TPR: True/sensitivity Positive Rate
